# Trusting the hand that feeds: microbes evolve to anticipate a serial transfer protocol as individuals or collectives

**DOI:** 10.1186/s12862-019-1512-2

**Published:** 2019-11-04

**Authors:** Bram van Dijk, Jeroen Meijer, Thomas D. Cuypers, Paulien Hogeweg

**Affiliations:** Theoretical Biology, Utrecht University, Padualaan 8, Utrecht, The Netherlands

**Keywords:** Experimental evolution, Predicting evolution, Serial transfer protocol, Resource cycle, Eco-evolutionary dynamics, In silico evolution, Digital microbes, Virtual microbes

## Abstract

**Background:**

Experimental evolution of microbes often involves a serial transfer protocol, where microbes are repeatedly diluted by transfer to a fresh medium, starting a new growth cycle. This has revealed that evolution can be remarkably reproducible, where microbes show parallel adaptations both on the level of the phenotype as well as the genotype. However, these studies also reveal a strong potential for divergent evolution, leading to diversity both between and within replicate populations. We here study how in silico evolved Virtual Microbe “wild types” (WTs) adapt to a serial transfer protocol to investigate generic evolutionary adaptations, and how these adaptations can be manifested by a variety of different mechanisms.

**Results:**

We show that all WTs evolve to anticipate the regularity of the serial transfer protocol by adopting a fine-tuned balance of growth and survival. This anticipation is done by evolving either a high yield mode, or a high growth rate mode. We find that both modes of anticipation can be achieved by individual lineages and by collectives of microbes. Moreover, these different outcomes can be achieved with or without regulation, although the individual-based anticipation without regulation is less well adapted in the high growth rate mode.

**Conclusions:**

All our in silico WTs evolve to trust the hand that feeds by evolving to anticipate the periodicity of a serial transfer protocol, but can do so by evolving two distinct growth strategies. Furthermore, both these growth strategies can be accomplished by gene regulation, a variety of different polymorphisms, and combinations thereof. Our work reveals that, even under controlled conditions like those in the lab, it may not be possible to predict individual evolutionary trajectories, but repeated experiments may well result in only a limited number of possible outcomes.

## Background

In order to see microbial evolution in action, we often rely on experimental evolution under controlled laboratory conditions. The Long-term Evolution Experiment (LTEE) [1] and similar shorter studies [2, 3] have, for example, evolved many generations of microbes using a serial transfer protocol, where microbes are repeatedly diluted and transferred to a fresh medium to start a new growth cycle. Conceptually, if we learn to understand how microbes adapt to such a resource cycle, we might one day be able to predict evolution in the lab and — ideally — also in nature. Indeed, a lot of evolution in the lab seems remarkably reproducible, where microbes show parallel adaptations both on the level of the phenotype as well as the genotype [4–11]. However, there also seems to be strong potential for divergent evolution, leading to diversity both between and within replicate populations [12–14]. Diversification events within populations in serial transfer regularly show cross-feeding interactions [12, 13, 15–17], where strains emerge that grow on metabolic by-products. These cross-feeding interactions are increasingly well understood with the help of metabolic modeling and digital evolution [18, 19]. A recent metagenomics study has revealed even more coexisting lineages in the LTEE than were previously reported [20]. It is however not yet clear whether all these polymorphisms are the result of uni-directional cross-feeding interactions, or if other mechanisms could drive coexistence in a simple experiment such as a serial transfer protocol. Furthermore, whether or not the diversified communities experience fundamentally different selection pressures and growth dynamics as a collective, is still an open question.

Prior to being subjected to lab conditions, the microbes used in the aforementioned experimental studies have all had a long evolutionary history in natural environments, experiencing harshly fluctuating and — more often than not — unfavourable conditions. While a serial transfer protocol at a first glance selects mostly for higher growth rates when resources are abundant (i.e. during the log phase), there is also selection to survive when resources are depleted and the population no longer grows (i.e. during the stationary phase). In fact, given the unpredictable conditions found in nature, some of the ancestors of *Escherichia coli* might have survived precisely because they diverted resources *away* from growth. Indeed, *E. coli* does exactly this during the stationary phase by means of the stringent response, regulating up to one third of all genes during starvation [21]. This response lowers the growth rate, but promotes efficiency and survival (i.e. a higher yield). While most microbes have ways to deal with starvation, the physiology of growth arrest varies a lot across different microbes, and especially display great variation in how long they can persist in the absence of nutrients (for an excellent review, see [22]). After prolonged starvation, many species of bacteria go through even more physiological changes, such as the GASP response [23], persistence [24], and sporulation [25]. Bacteria have also been shown to employ bet-hedging strategies with respect to these physiological changes [26–28], which could help to adapt to unexpected environmental changes. Finally, it has been shown that microorganisms can even adjust to *expected* environmental changes, anticipating regularity in environmental changes [24, 29, 30], which usually entails using predictive cues from the environment. All these responses, as well as other features that organisms have acquired during their evolutionary history (gene clustering, gene regulatory network architecture, metabolic regulation, *etc.*), might strongly influence the adaptation and reproducibility we observe in the lab today.

What do we expect when a complex, “pre-evolved” organism adapts to serial transfer protocol in the lab, given how clean and extremely regular these conditions are? We here use Virtual Microbes in order to firstly mimic natural evolution, acquiring Virtual “wild types” (WTs), which we then expose to a serial transfer protocol (see methods). We do so in order to obtain a fresh perspective on *whichgeneric adaptations* might appear in spite of evolutionary contingencies, and *how* these adaptations are achieved. We find that all the WTs — which are both genotypically and phenotypically diverse — evolve to anticipate the regularity of the serial transfer protocol by timing their growth rate, yield, and survival, to accurately fit the daily cycle. Yet, we observe many alternative paths in terms of growth dynamics trajectories, gene regulation, and diversification. Whereas some WTs adapt by means of clever gene regulation, others diverge into multiple strains with their own temporal niche, and others simply time their resource consumption as to not over-exploit the medium. In short, our WTs all recognized and exploited the regularity of the serial transfer protocol, having learned to trust the hand that feeds, but they solve this challenge by a variety of different mechanisms.

## Results

**Fig. 1:**
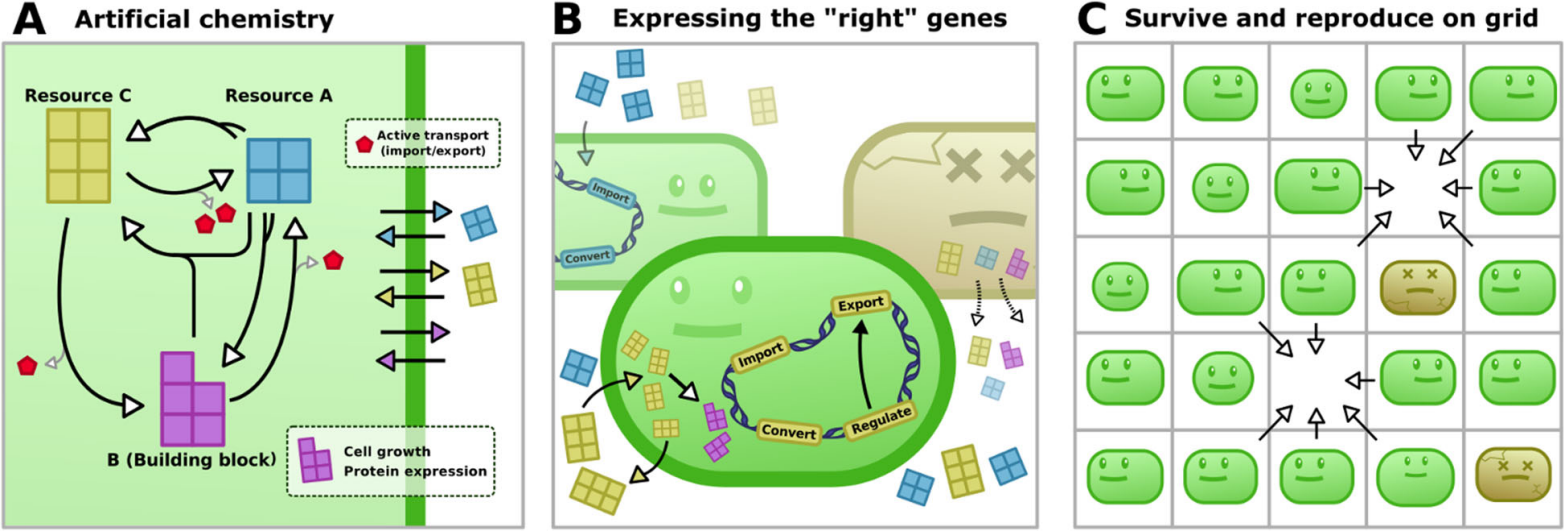
Virtual Microbes model overview. **a** At the basis of the Virtual Microbe model is an artificial “metabolic universe”, describing all the possible reactions that can be catalysed. Resources (yellow and blue) are fluxed in, but building blocks (purple) and energy (red) must be synthesized to express proteins and transport metabolites across the membrane, respectively. **b** A Virtual Microbe only needs to express a subset of all possible reactions to be viable, and no metabolic strategy is necessarily the “right” one. **c** The individuals grow and reproduce on a spatial grid, and can only reproduce when there is an empty spot. Death happens stochastically or when a cell has accumulated toxicity by having excessively high concentrations of metabolites. Since only cells that have grown sufficiently are allowed to reproduce, we simulate evolution with no prior expectation

In this study we use Virtual Microbes, a model of the eco-evolutionary dynamics of microbes (Fig.1 and methods). In short, the Virtual Microbe model is unsupervised, meaning that it aims to combine relevant biological structures (genes, genomes, metabolism, mutations, ecology, etc.), allowing us to study the emergent properties of fitness and evolution in an undirected system. In other words, by not explicitly defining what the model *should* do, we take a serendipitous approach to study microbial evolution. By modelling evolution with many degrees of freedom, the process can be seen as a “inventive” generator of attainable (and maintainable) adaptations [31], and can furthermore serve to debug false intuitions [32]. Our main objective in this study is to elucidate *generic adaptations* of evolution in a serial transfer protocol, to investigate how this is achieved, and to what extend it is constrained by prior evolution. In order not to lose track of the objective of *finding generic patterns*, we refrain from discussing and analysing every mechanistic detail, and instead focus on major observables and discuss some illustrative cases.

### Evolving Virtual Microbe “wild types”

**Fig. 2:**
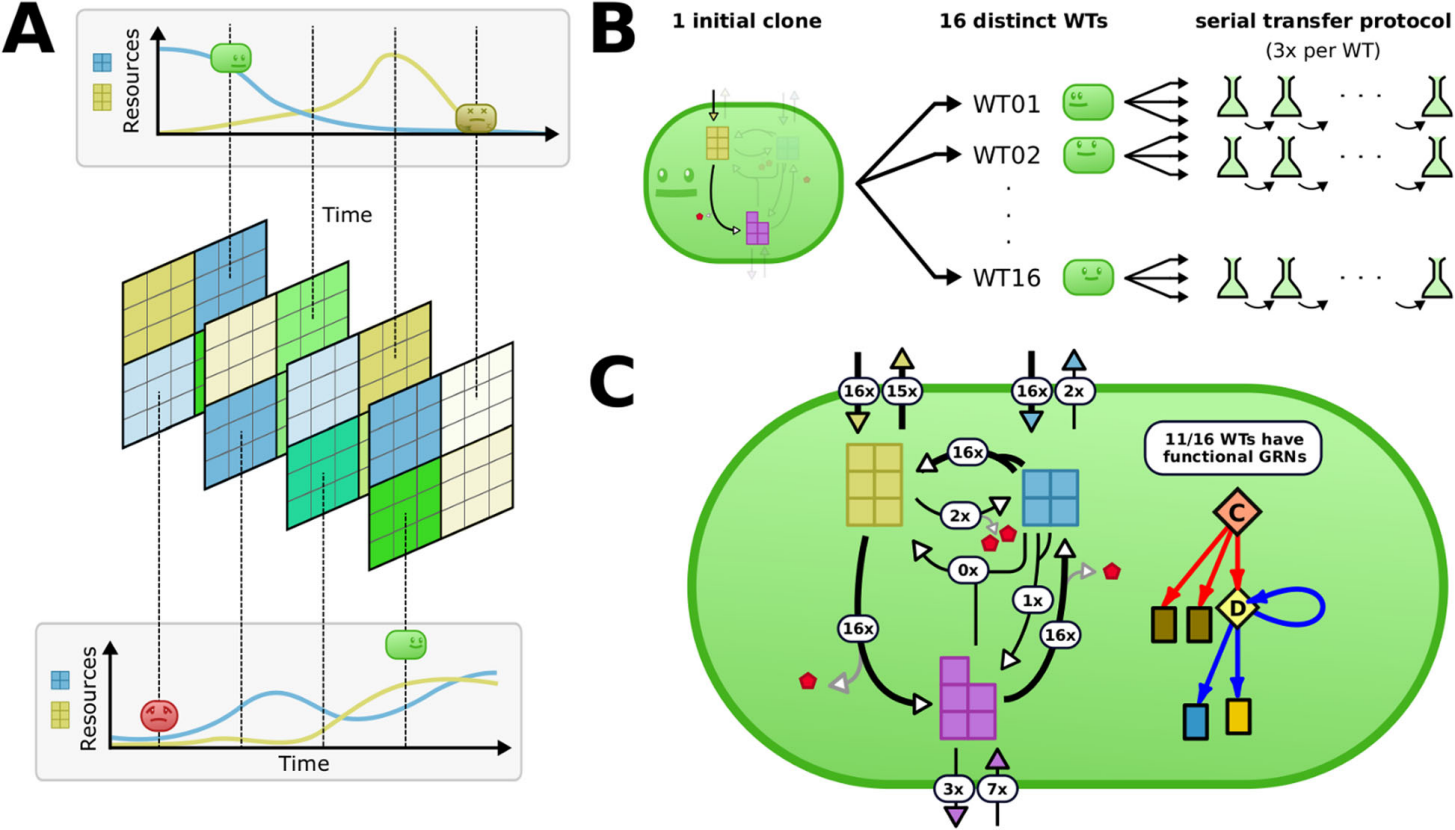
Evolution of Virtual “wild types” under naturally unpredictable and fluctuating resource conditions. **a** Natural evolution is mimicked by (harsly) fluctuating resource conditions, resulting in a wide variety of resource conditions. The (actual) grid is 40x40, with four 20x20 subspaces where the rates of influx vary stochastically. These subspaces do not impede diffusion of metabolites or reproduction. The fluctuations of the A and C resource (blue and yellow respectively) are independent, resulting in a variety of different conditions. **b** We repeat the evolution in natural conditions 16 times starting from the same (minimally viable) initial clone (varying the mutations that happen) yielding 16 distinct WTs. These WTs are later transfered to a serial transfer protocol. **c** In the white labels we show how many of the evolved WTs adapted to use particular reactions. The thicker arrows represent the shared core genome which consists of two resource importers, a metabolic cycle, and a C-exporter (yellow). Transcription factors (diamonds) were always present across WTs, but only 11/16 WTs visibly display changes in gene expression correlated with changes in the environment

Before evolving Virtual Microbes in a serial transfer protocol, we first evolved a set of Virtual “Wild Types” (WTs). Instead of optimizing these WTs solely for high growth rates or optimal metabolic flux, we here mimic natural circumstances by fluctuating resource conditions (Fig. 2a). When too little resource is available, the Virtual Microbes cannot grow, and can only stay alive for as long as their internal resources last. When too much resource is available however, the Virtual Microbes run the risk of accumulating too high concentrations of metabolites, resulting in increased death rates due to toxicity. Furthermore, a stochastic death process is implemented, allowing even a maximally flourishing Virtual Microbes to only live 100 time steps on average. To avoid extinction, we divided the total grid into four sub-grids, where the two resource metabolites A and C (yellow and blue in Fig. 1a) independently change in their influx rates with probability 0.01 (see Table 3). Thus, on average, an individual will experience one fluctuation in resource conditions during its lifetime (see full configuration in S1). While both influxed resources can be converted into building blocks required for growth, the rates of influx span four orders of magnitude (10^−5^ – 10^−1^, see Table 3), and conditions will thus vary from very favourable to very poor. Although poor conditions could cause a local population of microbes to go extinct due to limiting resources, total extinction is highly unlikely due to the 4 independent sub-grids. All this in turn depends on which resources the evolved Virtual Microbes like to consume (and at which rate), whether or not there is too much or too little resource, and whether or not space for reproduction is available. Finally, persisting in an unfavourable environment for a long time can be rewarding if conditions improve. All in all, this results in an unsupervised evolutionary process where there is no prior expectation of what metabolic strategy or gene regulatory networks might be best suited to survive. We study what will be the long-term target of the eco-evolutionary dynamics, not in terms of fitness, but in terms of what the Virtual Microbes evolve *to do*.

We evolved the same initial clone in the exact same “random” resource fluctuations, *only* varying the mutations that happened across ∼10.000 generations of evolution. This produced 16 distinct WTs with their own evolutionary history, which we then expose to the serial transfer protocol (Fig. 2b). Despite experiencing precisely the same fluctuations, no two WTs evolved to be the same. For example, we observe a great diversity in gene content, kinetic parameters of enzymes, gene regulatory networks and their complexity, and responses to environmental stimuli. The core metabolism is however strikingly similar across WTs, always consisting of a simple metabolic cycle. The rates of building block production and death rates are also very similar across all WTs (Additional file 1: Figure S3). In other words, it appears that there are many different ways to be fit, and that no solution is evidently better. The similarities and differences between our WTs are summarized in Fig. 2c, but we discuss this in more detail in Additional file 1: Section S1.

### In silico serial transfer evolution experiment

After evolving a variety of different WTs, we transfer the WTs to a serial transfer protocol. With regular intervals, all but 10 percent of the cells are removed, while at the same time refreshing the medium. Although time in Virtual Microbes has arbitrary units, we will refer to this process as the “daily” cycle from this point forward. Early in the day, during the log phase, high growth rates are very rewarding as there is a lot of opportunity to reproduce. However, once the population has reached stationary phase (having consumed all resources), it is favourable to survive and to not invest in growth any further. We will focus on how our WTs adapt to these alternating selection pressures. The results discussed here are found for a variety of different medium conditions (e.g. also see Additional file 1: Table S2). In the main text however, we present the 50 time step serial transfer protocol where the medium contained both resources (A and C), as this was a condition on which all WTs could be cultivated, ensuring equal treatment. We focus on the generic adaptations towards this protocol first, and then show how specific WTs and contingent factors from their evolutionary history shape these outcomes.

### All wild types evolve to anticipate the serial transfer protocol

**Fig. 3:**
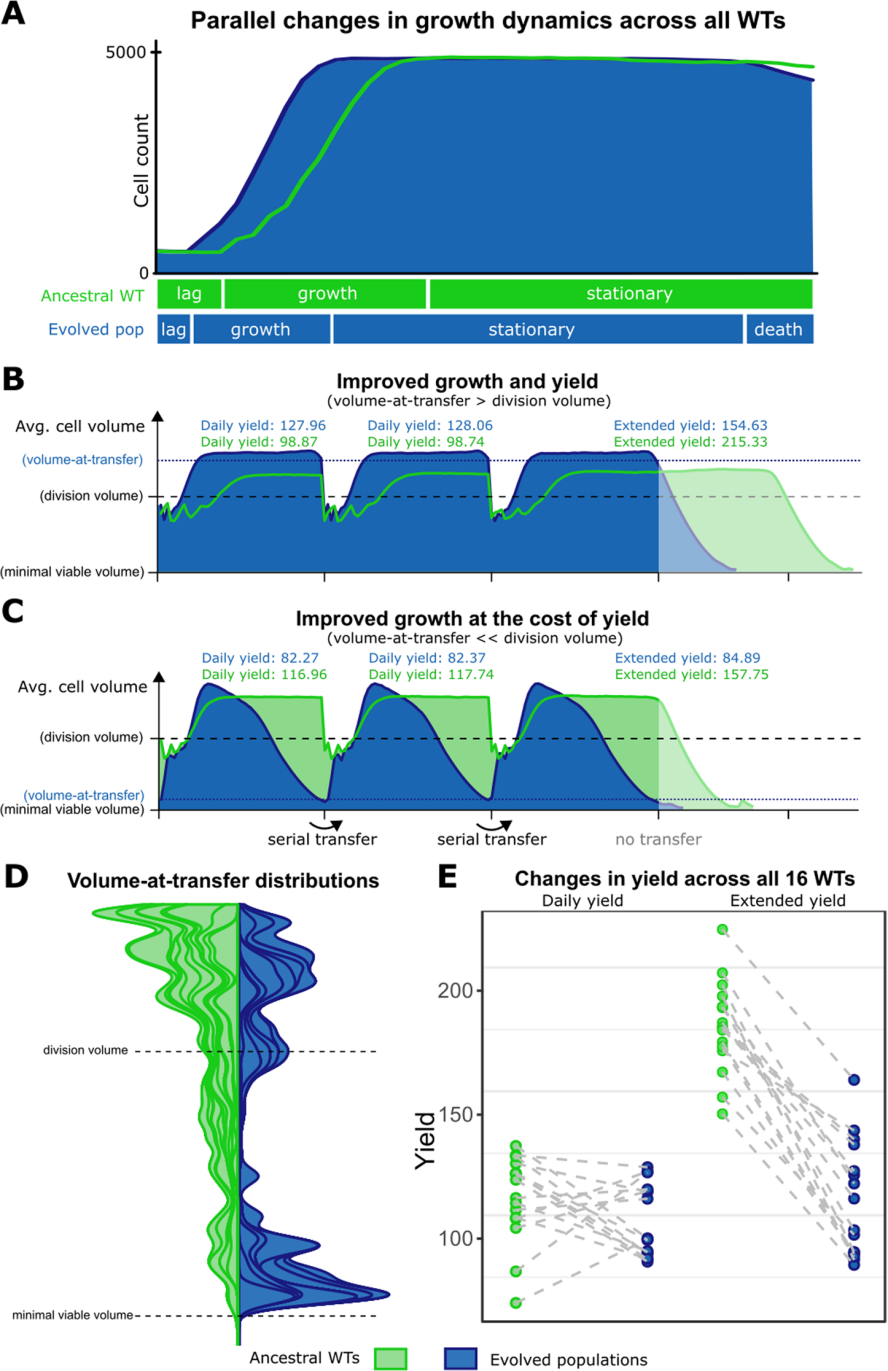
Virtual Microbes adapt to anticipate the regularity of a serial transfer protocol. **a** Growth dynamics of early population (green) and evolved populations (blue) in terms of cell counts. (WT03#1 taken as an illustrative example). **b-c** Two WTs (green) and the population after prolonged evolution in the serial transfer protocol (blue) are shown as an illustration of the anticipation effects. Over the course of 3 cycles, the average cell volume is plotted against time for the ancestral WT (green) and for the evolved population (blue). The y-axis (cell volume) indicates the minimal viable volume and division volume (which are fixed for the model), and the evolved volume-at-transfer (as measured at the end of the third cycle). Daily and extended yield are measured as defined in the method section. After the third cycle, serial transfer is stopped (transparent area), showing decreased survival of the evolved populations with respect to their ancestor. **d** Stacked density distributions are plotted for the volume-at-transfer both early (transfer 0-40, green) and late (transfer 760-800, blue). **e** The evolved changes in yield both “daily” (within one cycle of the protocol) and “extended” (after prolonged starvation) for all 16 WTs

After 800 days of evolving in a serial transfer protocol, we compare the ancestral WTs with the evolved populations. We first show some of the well-known growth dynamics of microbes: the lag-, log-, and stationary phase (Fig. 3a). As most experimental evolutionary studies in the lab, we too observe a decreased lag phase and an increased growth rate. The increased growth rate in the evolved population results in an earlier onset of the stationary phase, which therefore takes much longer than for their WT ancestors. Eventually, this leads to a phase where the cell count decreases again (death phase), revealing a decrease in survival for the evolved populations. To further study how this decreased survival comes about, we next investigated the dynamics of average cell volumes. Cell volume is an indicator for the “health” of the population, determining the ability to divide (minimal division volume) and survive (minimal viable volume). A first interesting observation is an increase in average cell volume during the log phase (Fig. 3b-c), which is also one of the first results from the LTEE [33]. However, after this increase in cell volumes during the log phase, evolved populations display a clear decrease in cell volumes, either at the end of the day (Fig. 3b), or during the whole stationary phase (Fig. 3c). Indeed, if we expose the populations to prolonged starvation by extending the day, the evolved populations die shortly after the anticipated serial transfer, while their WT ancestors survived for much longer (Fig. 3b-c, right-hand side). Strikingly, we observed that the cell volume at the time of transferring the cells to a fresh medium (henceforth ‘volume-at-transfer’) fall into two distinct categories. In the high yield scenario (Fig. 3b), cell volumes are maintained *above the division volume* until the very end of the day, whereas the low yield scenario, albeit having a higher growth rate, leads to a volume-at-transfer that is *just above minimal*. Indeed, the distribution of these observed volume-at-transfer across ancestral WTs are mostly high (Fig. 3d, left-hand side), while the evolved cells clearly show a bimodal distribution (Fig. 3d, right-hand side). Thus, all the populations evolved to either be ready to immediately divide at transfer (high yield mode), or exploit as much resource as possible while remaining above the minimal viable volume (high growth rate mode). Despite this difference in growth modes, both populations have evolved to accurately time the regularity of the serial transfer protocol. All evolved populations also show a consistent decrease in extended yield (Fig. 3e) relative to the WTs, as long term yield is now masked from natural selection. Finally, we found that this anticipation effect did not depend on details in the protocol, such as the length of the daily cycle or the number of resources used (Additional file 1: Figure S5 and Table S2). This reveals that a key selection pressure in a serial transfer protocol is not only growth as fast as possible, but also remaining viable until the next day, anticipating the next supply of nutrients.

### Evolution toward a growth-yield trade-off

**Fig. 4:**
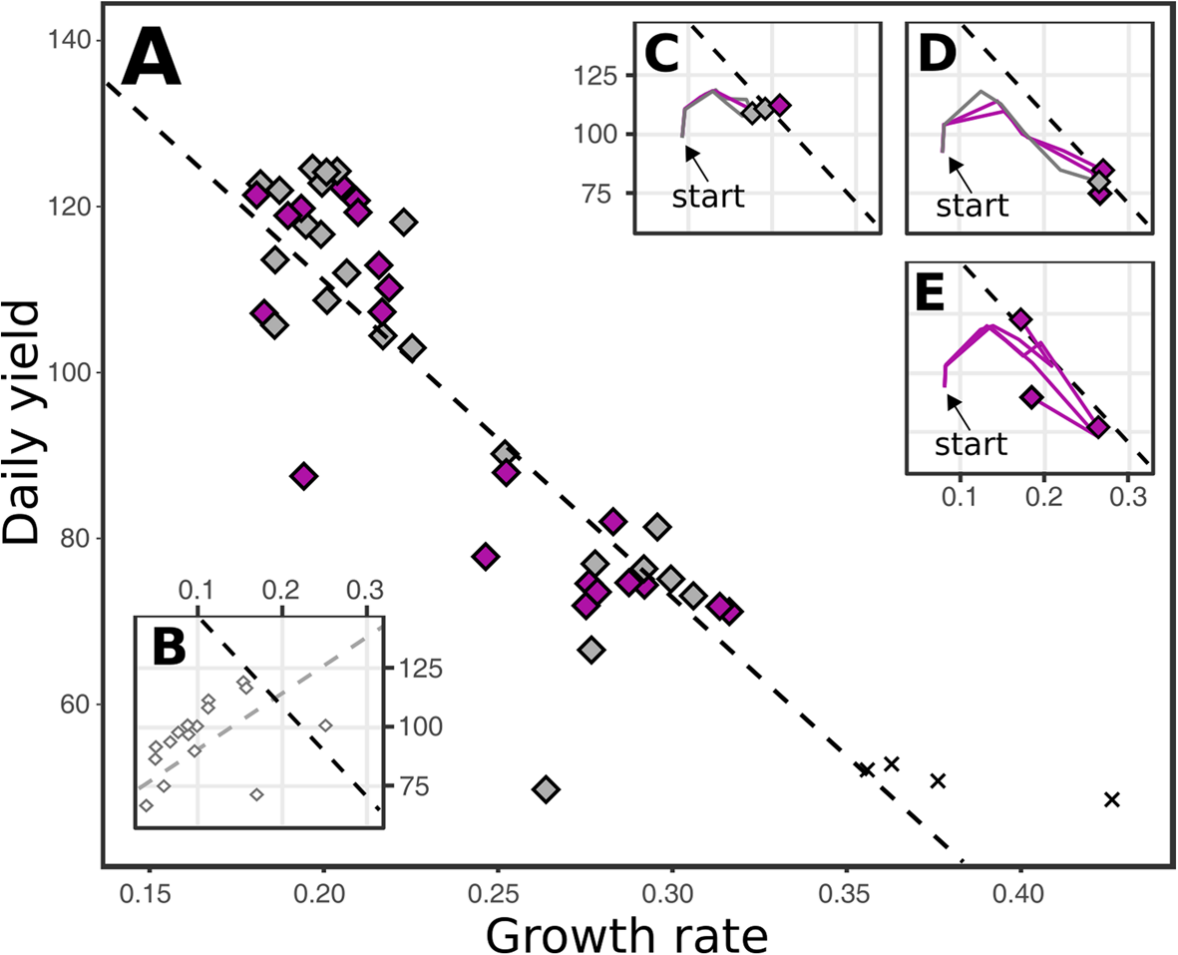
Trajectories towards a growth versus yield trade-off end in either the high growth rate mode or the high yield mode. **a** Growth rate (average building block production rate) is plotted against daily yield (average population biomass within a single cycle), for all the 48 experiments after adaptation to 800 serial transfers. The black dotted line is a linear regression model (R^2^ = 0.54). **b** Shows the initial points for all 16 WTs, which actually have a positive correlation between growth and yield (R^2^ = 0.32) instead of the negative correlation (black dotted line). **c-e** These insets display how the repeated evolution of certain WTs produce very similar trajectories towards the trade-off (time points are day 0, 20, 40, 100, 200 and 800), ending in either high daily yield (**c**) or low daily yield (**d**). Other WTs diverge after reaching the trade-off, and thus show more diverse trajectories when repeated (**e**). The colours of the end point symbols depict different modes of adaptation as discussed in the next paragraph (grey = no coexistence, purple = (quasi-)stable coexistence, black cross = extinction due to over-exploiting the medium)

The two extreme categories of cell volume dynamics from Fig. 3 illustrate a well-studied trade-off between growth and yield in microbial populations [34–36]. We next investigate how our different WTs evolve towards this trade-off, and how reproducible these trajectories are. For this, we repeated the serial transfer protocol 3 times for each WT, and follow the trajectories over time. After ∼800 serial transfers, all populations have adapted along a trade-off between growth and yield (Fig. 4a). No trade-off was not observed during the first cycle of the protocol, which instead shows a positive correlation between growth and yield (Fig. 4b), revealing how both growth and yield could initially be improved for most WTs. Evolution towards the trade-off, by improving both growth and yield by *e.g.* importing more resources or producing more building blocks, is similar across all WTs, although not all WTs approach it with the same angle (also see Additional file 1: Figure S6). Subsequent evolution on the trade-off diverges into two distinct clusters, representing the two aforementioned modes of high yield and high growth rate. This divergence is not only seen between different WTs (Fig. 4c-d), but also occurs in replicate experiments of the same WT (Fig. 4e, Additional file 1: Figure S6). Finally, specific WTs appear to more readily give rise to certain outcomes, having specific adaptations in their “mutational neighbourhood”. This is for example illustrated by two WTs (5 and 11) that repeatedly gave rise to mutants with extremely high, but unsustainable growth rates, causing populations to go extinct repeatedly (black crosses in Fig. 4). In summary, some WTs adapt in a similar way to the serial transfer protocol, while others (that have experienced the same amount of prior evolution) have diverging evolutionary trajectories and can reach different solutions, especially after having adapted towards the trade-off.

### Anticipating as a collective

**Fig. 5:**
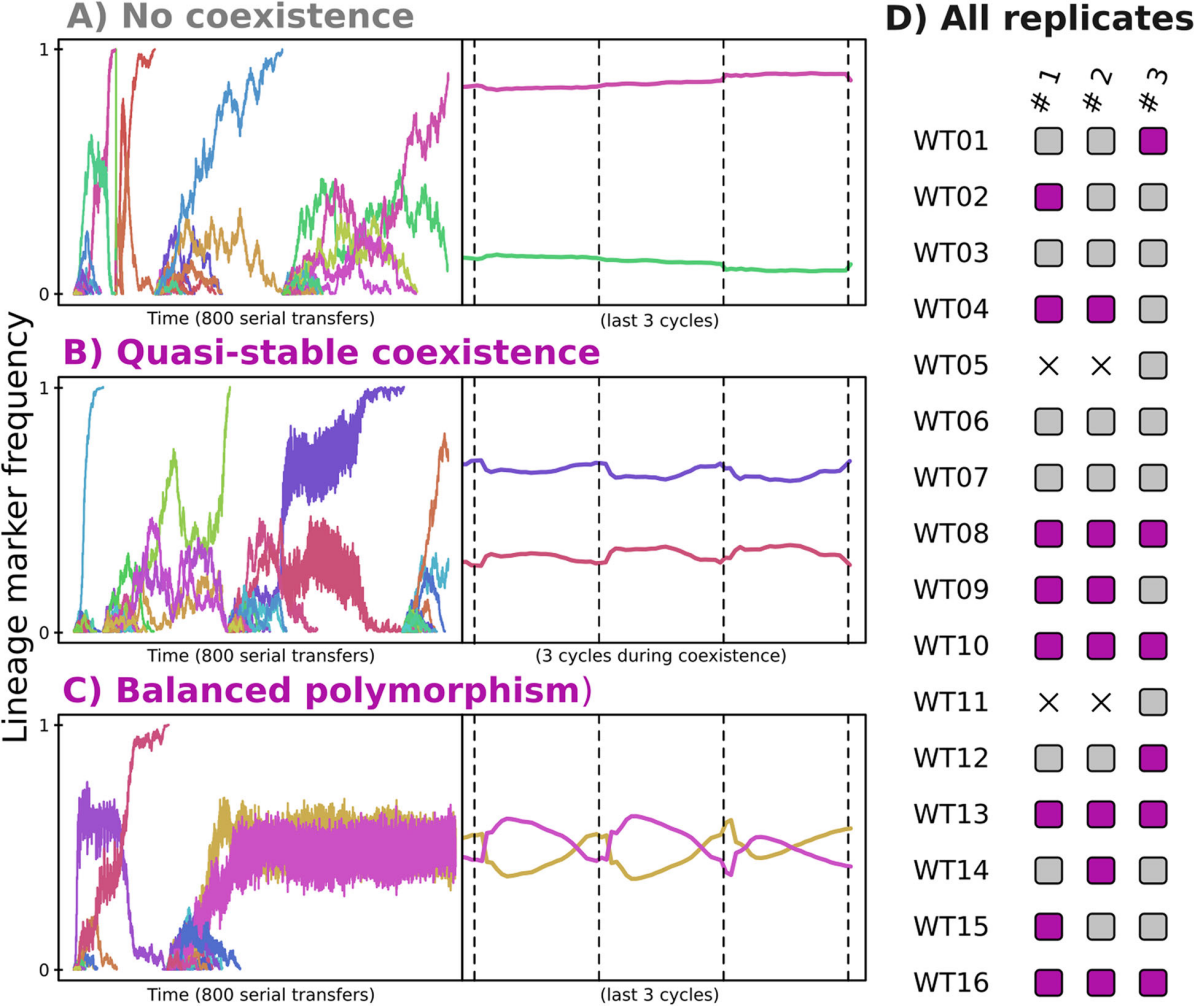
Dynamics of neutral lineage markers reveal balanced polymorphisms based on the daily cycle. **a-c** Neutral lineage marker (random colours) frequencies are plotted along 800 serial transfers (left hand side) and along 3 cycles. Panel A shows an example with no coexistence which is found in 23 out of 44 replicates, and panel B and C show (quasi-)stable coexistence, found in the remaining 21 replicates. **d** shows, for all 3 replicates of all WTs whether or not coexistence of neutral lineage markers was observed (grey = no coexistence, purple = (quasi-)stable coexistence, black cross = extinction due to over-exploiting the medium). Also see Additional file 1: Figure S8

So far we have only looked at population averages. Next, we study the dynamics of lineages and the evolved dynamics within cells. To track lineages we tag each individual in the population with a neutral lineage marker at the start of the experiment (analogous to DNA barcoding [37]). When a single lineage reaches fixation, we reapply these neutral markers, allowing us to quickly detect long-term coexistence. Moreover, these neutral markers allow us to study which arising mutants are adaptive in the different phases of the growth cycle. In Fig. 5a we show dynamics of neutral lineage markers that are frequently redistributed when one lineages fixates in the population, indicating that there is no long-term coexistence of strains. In contrast, Fig. 5b displays repeatedly observed (quasi-)stable coexistence, where two lineages coexist for some time, but coexistence was not stable in the long-term. Lastly, Fig. 5c shows stable, long-term coexistence, where the population sustains a balanced polymorphism until the end of the experiment. Based on these lineage markers (also see Additional file 1: Figure S8), coexistence (either quasi-stable or stable) was observed in 21 out of 44 extant populations (Fig. 5d).

By zooming in on the dynamics of coexisting lineage markers over a shorter time span (Fig. 5b-c, right-hand side), we can better understand how these lineages stably coexist. Notably, one lineage is dominating during log phase, while the other lineage performs better during stationary phase. In other words, the lineages have specialized on their own temporal niche. We find that these dynamics can be the result of three mechanisms (or combinations thereof): 1) cross-feeding on building block metabolites, 2) specialisation on either of the two resources, or 3) based on the growth vs. yield trade-off. Cross-feeding dynamics always resulted in quasi-stable coexistence (such as depicted in Fig. 5b), and never resulted in the balanced polymorphism as depicted in Fig. 5c), while the other two mechanisms (resource specialisation and growth vs. yield differentiation) most often resulted in long-term coexistence where lineages perform better together than they do alone (Additional file 1: Figure S9).

While specialisation on different resources is a well known mechanism for negative frequency dependent selection, it is far less evident how a growth vs. yield trade-off would result in a fully balanced polymorphism. Mutants with higher growth rates but elevated death rates have a very distinct signature of increasing in frequency early in the daily cycle and decreasing to much lower frequencies during the stationary phase (Additional file 1: Figure S7A), as apposed to lineages that increase in frequency throughout all phases of the cycle (Additional file 1: Figure S7B). While such mutants readily arise across our experiments, they often have difficulty rising to fixation due to the increased duration of the stationary phase, where they are unfit. In the meantime, a slower growing lineage with lower death rates can be optimized to utilize resources at low concentrations during stationary phase. These dynamics can give rise to a balanced polymorphism that does not depend on resource specialisation or cross feeding, and is also observed in our experiments with a single resource (Additional file 1: Table S2). Indeed, Fig. 5c illustrates how two lineages with more than a three-fold difference in death rates (±0.015 and ±0.048) can stably coexist.

discussed above can differ strongly across WTs and replicated experiments. For example, since de novo gene discoveries were disabled during this experiment, cross-feeding on building blocks is only possible if the ancestral WT had the necessary importer for building blocks, which was true only for 6/16 WTs. Similarly, even though all WTs have the necessary importers for both the A and C resource, one WT consistently diverged into an A- and C-specialist (WT10). While other WTs have multiple gene copies for these importers, WT10 had only 1 copy of both genes, making the loss-of-function mutations readily accessible. In conclusion, although all polymorphic populations also anticipate the serial transfer protocol, they do so in a different way than populations consisting of a single lineage. They all consist of strains which time growth and survival strategies in relation to each other in order to precisely finish the available nutrients by the end of the day.

### Individual anticipation by tuning and trimming the gene regulatory network

**Fig. 6:**
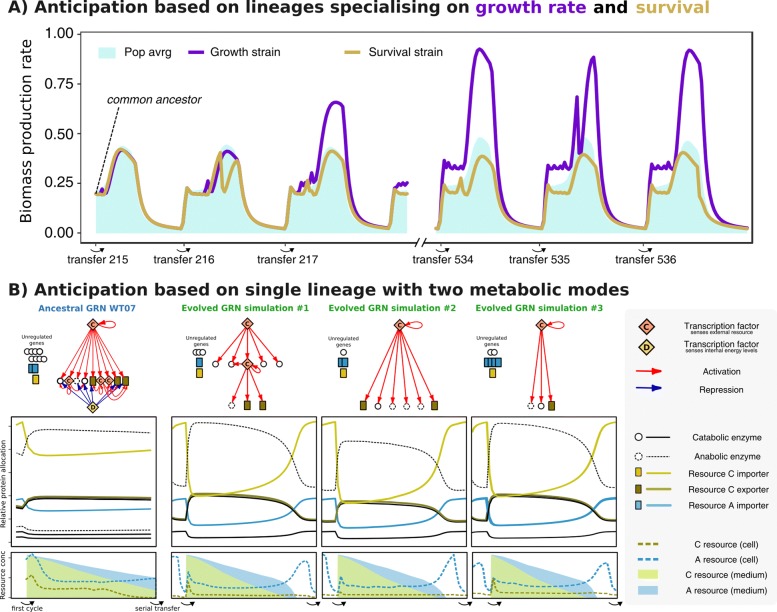
Anticipation can entail polymorphism or a single lineage that switches between two metabolic modes. **a** Two lineages occupy different niches on the growth vs. yield trade-off WT02#01 diverges into a slow growing lineage (yellow lineage, average death rate ±0.015) and a faster growing lineage with elevated death rates (blue lineages, average death rate ±0.048), together anticipating the serial transfer protocol. **b** A single lineage anticipates the daily cycle by trimming and tuning the gene regulatory network. On the left the ancestral GRN, protein allocation dynamics, and resource concentrations are displayed over the course of 1 day. Next, after 400 days, all three independent simulations of WT07 are shown to have evolved to anticipate as a single lineage with two metabolic modes

The previous section illustrates how multiple lineages can coexist because the predictable serial transfer protocol produces temporal niches. However, many of our WTs do not show any tendency to differentiate like this, and instead always adapt to the serial transfer protocol as a single lineage (Fig. 6d). In order to better understand this, we will now look at the intracellular dynamics of WT07, and how it changes when adapting to the protocol. WT07 is one of the more “clever” WTs with a relatively complex GRN, and displays strong responses in gene expression when exposed to fluctuations. In Fig. 6b we show that WT07 consistently adapts to the protocol by switching between two modes of metabolism, where importer proteins are primed and ready at the beginning of the cycle, and exporter proteins and anabolic enzymes are suppressed during stationary phase. Despite some differences in the structure of the evolved GRNs, the protein allocation patterns are virtually indistinguishable across the three replicate evolutionary experiments. Interestingly, although no parallel changes were observed in the kinetic parameters of proteins, we do observe the parallel loss of an energy-sensing transcription factor as well as increased sensitivity of the TF that senses the external resource C. In other words, even though all mutations are equally likely, evolution apparently happened mostly through loss, and tuning and trimming of the GRN. Modulation between two metabolic modes allows this single lineage to switch between log and stationary phase, occupying both temporal niches. Indeed, a second lineage never appeared for this WT (Fig. 6b and Additional file 1: Table S2).

### Individual and collective solutions have similar macro-level observables

**Fig. 7:**
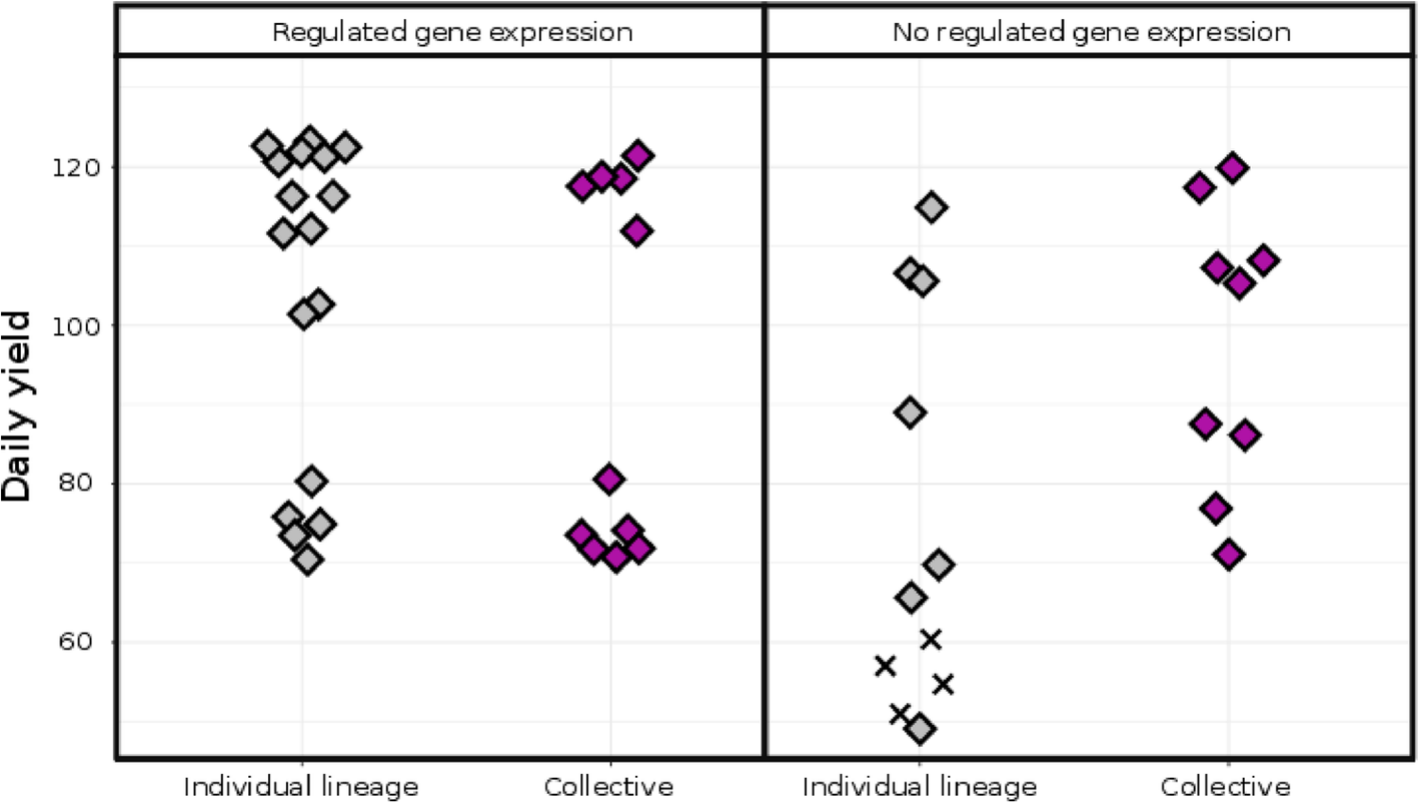
Individual and collective solutions have similar macro-level observables The daily yield for all the evolved populations is shown, for groups of individual / collective solutions with and without regulated gene expression. Colours and symbols are identical to previous figures (grey=no coexistence, purple=coexistence). Only the non-regulating, individual lineages perform significantly worse than any of the other groups (performing all 6 Wilcoxon rank-sum tests with *α* 0.05)

We have illustrated how all of our evolutionary experiments result in two modes, one with high yield, and another with high growth rates and lower yield. We have also shown how populations could or could not diversify into two strains, and how certain populations used regulated gene expression to adapt to all growth phases by itself. The four different combinations of collectives vs individual and regulating vs. non-regulating solutions, and their daily yield, are shown in Fig. 7. As can be seen, all these combinations anticipate the serial transfer protocol using either the high yield or high growth rate strategy, and achieve similar values. The non-regulating individual solutions however clearly perform more poorly, as these populations lack the ability to fill both temporal niches (note that gene discoveries are disabled during the serial transfer experiment, so gene regulation cannot evolve *de novo*). Also note that, although the regulating WTs could fill both temporal niches by themselves, this does not prevent balanced polymorphisms from forming repeatedly. These results show that either a collective solution and/or gene regulation is required to be well-adapted to a serial transfer protocol, and that which solution is used is not observable on the overall macro-level.

## Discussion

In this study we have taken a serendipitous approach to study how microbes adapt to a serial transfer protocol, and to what extent this is determined by their evolutionary history. The Virtual Microbe modelling framework serves this goal by building biology from the bottom up, *i.e.* implementing basic biological features and their interactions. We observe that regardless of their evolutionary history, all WTs learn to anticipate the regularity of the serial transfer protocol by evolving a fine-tuned balance between high growth rate and yield. Long-term survival without nutrients, which is now masked from natural selection, always deteriorates after prolonged exposure to such a protocol. Furthermore, this anticipation is done in two distinct ways. The high yield mode makes sure that the cells are ready to divide as soon as transferred to a fresh medium, whereas the high growth rate mode maximally exploits the medium but results in a poor performance during the stationary phase. We next show that WTs have similar trajectories towards a growth versus yield trade-off, but may subsequently diverge along it. Polymorphisms within populations are frequently observed, which can happen by means of cross-feeding interactions, resource specialisation, or by means of growth vs. yield specialisation. We furthermore find that these evolved collectives are dependent on one another, as both lineages perform better in the presence of the other. Finally, we show that regulated gene expression allows for an individual lineage to fill both temporal niches by itself, but that populations without regulated gene expression can still be well adapted to the protocol by diverging into two strains. In general, our results are robust to details in the serial transfer protocol, such as using only a single resource, or varying the interval between transfers (see Additional file 1: Table S2). The anticipation effects therefore appear to be generic features of microbes exposed to prolonged evolution in a serial transfer protocol.

How do our results map onto experimental evolution in the lab? *E. coli* REL606 has been subjected to a daily serial transfer protocol for over 30 years (∼70.000 generations) in the LTEE. Many of our observations are very similar to the LTEE, such as the improved growth rate and cell sizes during the log phase[33], the (quasi-)stable dynamics of coexisting lineages[20], and “leapfrogging” dynamics (e.g. Fig. 5a-b) where an abundant lineage is overtaken by another lineage before rising to fixation [38, 39]. The comparison with respect to the growth rates, yield, and the anticipation effects discussed in this work, is however less straightforward. We have observed how all our WTs quickly evolve to be maximally efficient given our artificial chemistry, and only subsequently diverge along the apparent growth versus yield trade-off (see Additional file 1: Figure S6). In the LTEE, growth and yield have continued to improve so far, and although a trade-off has been observed *within* the populations[40], no growth versus yield trade-off between the replicate populations has been observed yet. Nevertheless, we propose that anticipation of periodic environmental change, and a growth versus yield trade-off, provides testable hypotheses for the LTEE and similar experimental studies.

More similarities with empirical studies are found in the surprising number of experiments that result in balanced polymorphisms. A repeatedly observed mechanism for such a polymorphism is cross-feeding [11, 13, 16, 17], where modeling has shown that this adaptive diversification involves character displacement and strong niche construction[18], and furthermore strongly depend on the regularity of a serial transfer protocol [19]. We however also found balanced polymorphisms that did not include cross-feeding, involving one lineage with high growth rates during log phase and a slower growing lineage which performs better in stationary phase. Similar mechanisms of coexistence has been observed in respiratory and fermenting strains of *Saccharomyces cerevisiae* in chemostat [34], and single nucleotide mapping has furthermore revealed the existence of this trade-off [35]. These results are directly related to r/K selection theory [41], which describes an inherent conflict between the quantity and quality of ones offspring. Indeed, these dynamics have been shown to lead to two species stably coexisting in microbial populations [36, 42, 43]. Manhart & Shakhnovich [44] furthermore show that an unlimited number of species can theoretically coexist within a serial transfer protocol, occupying any niche on a trade-off continuum. Here we show that these dynamics can emerge from a more complex eco-evolutionary setting. However, our results suggest that the trade-off between growth and yield is not continuous, as intermediate solutions rarely evolve. This is caused by the fact that as soon as the volume-at-transfer for our digital microbes is smaller than the division volume (i.o.w. something else than the main nutrient becomes limiting for division), a cell may as well exploit its resources fully.

Experimental evolution of *Pseudomonas fluorescens* has shown that different evolutionary paths can lead to the same phenotypic adaptations in a new environment [45, 46]. On the other hand, many studies have also suggested that adaptation can often entail mutations in the same genes [47, 48]. In our experiments, prior adaptations can in some cases strongly shape the way subsequent evolution plays out, but these evolutionary constraints can strongly differ between WTs (Additional file 1: Figure S6). Furthermore, these data show that these evolutionary constraints may or may not diminish after prolonged evolution. There is a lot of variation on the predictability during the serial transfer experiment, revealing that evolutionary constraints by means of historical contingencies, are themselves the result of contingencies.

A factor that has been hypothesised to strongly impact the predictability and evolvability of biological systems are their GRNs [6, 49–51], where for example global transcription factors could serve as mutational targets with large-scale phenotypic effects [8]. While our results (Fig. 6b) clearly show an example where similar mutations result in similar adaptive changes, other regulating WTs showed much less predictability. For example, WT #09 is another strong regulating WT, but showed different outcomes with respect to diversification and regulation in all 3 cases. In other words, while the GRN appears to add knobs and buttons for evolution to push, other mechanisms are clearly available to adapt and be fit in a serial transfer protocol. One such mechanism could be ‘metabolic regulation’, which has recently been shown to be able to achieve very high levels of robustness without leading to a loss in adaptive degrees of freedom [52]. Because all the kinetic parameters of enzymes (K _*m*_, V _*max*_, etc.) in the Virtual Microbes are freely evolvable, it is likely that this metabolic regulation of homeostasis plays a very important role in Virtual Microbes. This could furthermore explain why the differences in evolvability between regulating and non-regulating populations were smaller than we initially expected. We have indeed observed that, for certain WTs, a change in metabolism could bypass regulated protein expression by means of kinetic neofunctionalistaion of importer proteins, that evolved to be sensitive to different concentrations. Although such a solution does waste more building blocks on the continuous production of importer proteins, it is also much more responsive to environmental changes. It is possible that subtle differences like this explain, for example, why two of our WTs were much more sensitive to extinction by over-exploiting the medium than others. Furthermore, although the phenotypes that are reachable can be limited by prior evolution [53], the trajectories of evolution may be much less predictable on the long-term [54]. The role of metabolic regulation, and how this interplays with the repeatability and timescales of evolution, is a promising endeavour for future studies.

### Who is anticipating what?

Our experiments reveal how populations of microbes can evolve to anticipate the regularity of a serial transfer protocol, trusting that new resources will be delivered on time. The concept of microbial populations anticipating predictable changes is frequently observed in nature [29, 29, 55], and is supported by theoretical models [30, 56]. This form of anticipation however typically entails an environmental cue, where a preceding unrelated signal is used to anticipate environmental changes, usually followed by individuals taking some form of action. Without the necessity of such a cue, we show that anticipation can readily emerge in many different ways from an eco-evolutionary process. Although our form of anticipation is more passive, where not an individual but the system as a whole has temporal dynamics that accurately fit the protocol, this does not necessarily exclude individual-based anticipation. Like WT#07, most of the evolved regulating populations actually did not evolve to down-regulate their resource importers during the stationary phase, despite having repeatedly evolved to down-regulate other catabolic and anabolic enzymes (illustrated in Fig. 6b). Since no more resource is available, and building blocks are consumed in order to keep expressing these importer proteins, this clearly does not have a positive impact during the late stationary phase. One can wonder why these individuals seem to keep the engine running. Whereas bet-hedging strategies have been shown to be a way to deal with irregular environmental changes [24, 26–28, 57, 58], this passive form of anticipation can be a way deal with regular, predictable changes in the environment. Furthermore, this could potentially be the first step towards active anticipation by means of a circadian rhythm, such as the sunflower heliotropism [59] and the diurnal migration of life in lakes and oceans [60–62].

### Moving towards an eco-evolutionary understanding

The dynamics of Virtual Microbes expose that even a simple serial transfer protocol entails much more than sequentially evolving higher and higher growth rates. Instead, adaptation is an eco-evolutionary process that strongly depends on prior evolution, timescales, the presence of other competitors and mutants, and transient fitness effects. Although we found that competition experiments generally favoured the evolved population over the ancestral WTs, there were exceptions to this rule. It is therefore possible that the ancestral WTs perform better in such an experiment, but that this does not describe the stable eco-evolutionary attractor. Indeed, survival of the fittest is an eco-evolutionary process where any emerging lineage interacts with other lineages (or with other mutants) through changes in the environment, often resulting in a collective, community-based solution rather than the winner of all pair-wise interactions [44]. Furthermore, faster growth becomes less and less important as populations become better adapted to the serial transfer protocol, perhaps making the aforementioned interactions between lineages increasingly relevant. Other recent studies have recently elucidated the importance of eco-evolutionary dynamics [44, 63], and how this can readily give rise to coexistence of multiple strains which could not have formed from a classical adaptive dynamics perspective [64, 65]. Indeed, metagenomics have revealed much more diversity in the LTEE than previously anticipated [20]. Shifting focus from competition experiments towards the ever-changing selection pressures that emerge from the eco-evolutionary dynamics and interactions, will make the field of experimental evolution harder, but more intriguing, to study.

## Conclusions

We have studied how in silico WTs of Virtual Microbes adapt to a serial transfer protocol like that of the LTEE. The LTEE has shown a sustained increase in competitive fitness, and intensive research displays how the evolved clones are still improving their growth rates with respect to their ancestor as to this day [66–68]. Our experiments have generated a novel hypothesis that microbes in a serial transfer protocol will eventually evolve to anticipate the regular resource interval, and can do so by evolving either a high growth rate mode, or a high yield mode. Both these modes can be achieved by a single individual lineage, or by a collective of two strains which both have their own temporal niche. Taken together, our results reveal important insights into the dynamics and relevant selection pressures in experimental evolution, advancing our understanding of the eco-evolutionary dynamics of microbes.

## Methods

A full description of the model and underlying equations is available online (https://bitbucket.org/thocu/virtual-microbes and https://virtualmicrobes.readthedocs.io). Here we summarize the sections of these documents that are relevant to this study.

### Finding generic patterns of evolution

Experimental evolution is, of course, done on organisms that have evolved for a long time under a wide variety of conditions. These studied organisms all have their own evolutionary history, and differences in how they deal with starvation, stress, changes in resource etc. With Virtual Microbes we are able to evolve a de novo set of “wild types” (WTs), adapted to live in such severely fluctuating resource conditions. We can then explore how these WTs adapt to experimental evolution, and find generic patterns of evolution. To find generic patterns without being biased towards specific solutions, the biology of Virtual Microbes build-up from many levels with many degrees of freedom. One downside of this strategy can be that it can be hard for readers to understand all the underlying assumptions and algorithm and that many simulations result in a slightly different anecdote. However, we encourage the reader to read this work as though reading about ‘real’ biological evolution, where the experiments reveal new generic patterns and generate new hypotheses. With or without an understanding of the mechanistic details, relatively simple multilevel models can capture the eco-evolutionary dynamics of microbes, allowing us to study what happens, what else emerges from these dynamics “for free”, and equally important: what needs further explanation?

### Model overview

Virtual Microbes metabolise, grow and divide on a spatial grid (Fig. 1c). Here, we use two parallel 40x40 grids with wrapped boundary conditions. One grid contains the Virtual Microbes and empty grid-points, and the other describes the local environment in which the Virtual Microbes live. This environmental layer holds influxed metabolites, waste products of Virtual Microbes, and spilled metabolites from lysing cells (Fig. 1b). In order to express proteins, grow, and maintain their cell size, Virtual Microbes must synthesize predefined metabolite(s), which we call building blocks. These building blocks are not directly provided, but must be synthesized by the Virtual Microbes by expressing the right proteins, allowing them to pump metabolites into the cell, and convert metabolites into one another (Fig. 1a). The expression of these proteins depends on genes on genomes that undergo a wide variety of possible mutations upon reproduction (Table 1). Genomes are circular lists of genes, each with their own unique properties (e.g. K _*m*_, V _*max*_ for enzymes, K _*ligand*_ and binding motif for TFs). The level of expression is unique for each gene, and is determined by its evolvable basal transcription rate and how this rate is modulated by transcription factors. When an enzyme or transporter gene is expressed, that specific reaction will take place within the cell that carries that gene. Note however that in the complete metabolic universe, many more possible reactions exist. The genome of an evolved Virtual Microbes will typically only use a subset of all the possible reactions. Genes to catalyse new reactions and novel TFs can be discovered through rare events. Which genes end up being selected for is not explicitly defined, but the result of a birth and death process. Birth depends on the availability of empty space and resources to synthesize new building blocks, whereas death depends on the ability to survive under a variety of different conditions and the potential accumulation (and avoidance) of toxicity. The resulting survival of the fittest (referred to as “competitive fitness” by Fragata et al., 2018) is an emergent phenomenon of eco-evolutionary dynamics[69].

**Table 1:** Types of mutations and their probabilities in WT evolution and serial transfer protocol (STP)

Mutation	Description	Prob (WT evolution)	Prob (STP)
Duplication	A stretch of 1 or more genes is duplicated in tandem	0.005	0.0015
Deletion	A stretch of 1 or more genes is deleted	0.005	0.0015
Inversion	A stretch of 1 or more genes is inverted in order	0.005	0.0015
Translocation	A stretch of 1 or more genes is moved to a random location	0.005	0.0015
(stretch length)	Geometrically distributed with p = 0.3	-	-
Gene discovery	Per time-step probability of discovering a new (randomly parameterised) gene.	0.0002	(disabled)
HGT	Per time-step probability of copying a gene from a cell closeby	0.002	(disabled)
Point mutation	Per gene per generation probability of modifying a single parameter of a gene (promoter strength, Michaelis Menten constants)	0.005	0.0015
Regulatory mutation	Per gene per generation probability of (partially) modifying the upstream binary operator sequence of a gene	0.005	0.0015

**Table 2:** Gene level mutations and the boundary conditions

Parameter	Gene types	Value range in simulation
Promoter strength	Enzyme, transporter, TF	[0.001,10]
*K*_*substrate*_	Enzyme, transporter	[0.001,10]
*K*_*energy*_	Transporter	[0.001,10]
*K*_*ligand*_	TF	[0.001,10]
*K*_*operator*_	TF	[0.001,10]
*V*_*max*_	Enzyme, Transporter	[0.001,10]
effect-bound	TF	[0.001,10]
effect-apo	TF	[0.001,10]
ligand	TF	A, B, C, or e
exporting	Transporter	True,False
sense-external	TF	[True,False]
binding-motif	TF	bit flip at random position
operator-sequence	Enzyme, Transporter, TF	bit flip at random position

**Metabolic universe** The metabolic universe in Virtual Microbes is an automatically generated (or user defined) set of metabolites and reactions between them. The simple metabolic universe used in this study was automatically generated by a simple algorithm that defines 4 classes of molecules, how they can be converted into one another by means of 6 reactions, how fast they degrade, diffuse over the membranes, *etc.* (see Table 4).

**Table 3:** Grid setup and environmental forcing in WT evolution and serial transfer protocol (STP)

Option (WT evolution)	Description	Value or range
Maximum population size	As defined by the size of the grid (40x40)	4900
Sub-grids	The grid is sub-divided into n grids where fluctuations are independent	4
Fluctuation frequency	Probability (per time step) of 1 metabolite (A or C) changes in influx in one of the sub-grids	0.01
Fluctuation range	New influx of metabolite is sampled uniformly from range	[10e-5, 10e-1]
Extracellular metabolite outflux	Rate at which metabolites outside of cells wash out	0.01
Option (serial transfer protocol)	Description	Value or range
Maximum population size	As defined by the size of the grid (70x70)	4900
Number of cells serially transferred	A (near) tenfold dilution of cells	500
Time steps of cycle	This represents, for example, the“ 24 h” serial transfer protocol of the LTEE	50 (AUT)
[*A*] at beginning of cycle	Amount of resource A given at the beginning of the cycle	1.25
[*C*] at beginning of cycle	Am mount of resource C given at the beginning of the cycle	1.25
Extracellular metabolite outflux	Assuming metabolites can no longer wash out of the system	0.0

**Table 4:** A priori defined metabolites and reactions in artificial chemistry

Metabolite	Mass	Class	Degradation rate	Diffusion rate	Toxicity level
A	4	Resource	0.01	0.02	0.2
B	5	Building block	0.1	0.0015	0.2
C	6	Resource	0.01	0.015	0.2
e	1	Energy carrier	0.5	0.0015	0.2
Potential reactions (6)					
1C → 1B + 1e	1C → 1A + 2e	1A + 1B → 1C	2A → 1C	2A → 1B	1B → 1A + 1D

The metabolism is simulated on the grid in terms of Ordinary Differential Equations (ODEs) using the Gnu Scientific Library in Cython. These ODEs include the influx of molecules into the system, transport or diffusion across the membrane, intracellular metabolism (including expression and decay of proteins), biomass production, cell volume, the build-up of toxicity, etc.. Diffusion between grid points was implemented as a simple local diffusion process, and is interleaved with the ODEs for efficiency. The number of simulations was limited to 16 WTs and 16x3 “lab” experiments due to computational feasibility. Statistics in this study only report effect sizes, as p-values are irrelevant in simulated studies [70].

**Transmembrane transport** For all molecules, transporters exist that import or export molecules across the cell membrane. Michaelis-Menten kinetics determine the transmembrane transportation with rate *v* :

$$v = {v_{{max}_{\mathcal{T}}}} \cdot [\mathcal{T}] \cdot \frac{[S] \cdot [e] }{([S] + K_{S}) \cdot ([e] + K_{e}) } $$

where $\mathcal {[T]}$ is the concentration of the transporter protein, [*S*] is the concentration of substrate transported, and [*e*] is the concentration of available energy carrier metabolites. *K*_*S*_ and *K*_*E*_ are the Michaelis-Menten constants for the substrate and energy carrier respectfully. Depending on the direction of transport (importing or exporting) [*S*] is either the external or the internal concentration of the substrate. Note that for any gene on the genome of a Virtual Microbe, $V_{max\mathcal {T}}, K_{S}$ and *K*_*E*_ are all freely evolvable parameters.

**Metabolism** Similar to the transport, metabolic rates are catalysed by proteins by Michaelis-Menten kinetics with rate *v*:

$${\kern29pt}v = {v_{{max}_{\mathcal{E}}}} \cdot [\mathcal{E}] \cdot\frac{\prod_{R\in \mathcal{R}} [R] }{\prod_{R\in \mathcal{R}} ([R] + K_{R}) } $$

where [$\mathcal {E}$] is the concentration of the enzyme catalysing the reaction, $\mathcal {R}$ the set of all reactant metabolites, and *K*_*R*_ and $v_{{max}_{\mathcal {E}}}$ are evolvable kinetic parameters of enzyme $\mathcal {E}$.

**Biomass production** Virtual microbes convert building block *B* to a biomass product *P*, which is consumed for cell growth and maintenance *Growth*(*B*) and protein production *Prod*(*B*), and determines strength with which individuals compete to reproduce. Biomass is next converted to cell volume with a fixed rate, and used for protein expression depending on the demands by the evolved genome. In other words, high rates of expression demand more biomass product for proteins, leaving less biomass product to invest in cell volume or maintenance (see cell volume growth). In total, the rate of change of *P* then becomes

$${\begin{aligned} \frac{dP}{dt} &\,=\, Production(B) - Growth(B) - Protein expression(B) \\&- dilution - degradation \end{aligned}} $$

where B is the concentration of building block metabolites. Production is a linear conversion of B into P, whereas growth, protein expression, and dilution depend on the dynamics of the cell. Biomass product is then consumed by cellular growth and protein expression which are a function of the building block concentration, is diluted proportional to the changes in cell volume, and degradation is fixed. Consumption for protein expression is summed over all genes:

$$\sum_{i=1}^{N_{genes}}{Pr_{i}\cdot {Reg}_{i} } $$

where *Pr*_*i*_ is the basal expression rate of gene *i*, either up or down-regulated if transcription factors are bound to its operator sequence *Reg*_*i*_ (see transcriptional regulation).

**Cell volume growth** We assume that cell volumes a maximum cell size *MaxV* and that there is a continuous turnover *d* of the cell volume at steady state, ensuring the necessity to keep on metabolising even if there is no possibility to reproduce (i.e. if the grid points are all full). Volume then changes as

$$\frac{dV}{dt} = g \cdot V \cdot \frac{1-V}{{MaxV}} -d \cdot V $$

**Transcriptional regulation**The rates at which genes are expressed is a function of the basal expression rate of the gene and the concentrations of binding TFs and their molecular ligands. The intrinsic basal expression rate of a gene is encoded by a strength parameter in a gene’s promoter region. This basal expression rate can be modulated by TFs that bind to an operator sequence associated with the gene. Binding sites and TF binding motifs are modelled as bit-strings and matching depends on a certain fraction of sequence complementarity. If a minimum complementarity is chosen <1 a match may occur anywhere within the full length of the operator binding sequence and the TF binding motif. The maximum fraction of complementarity achieved between matching sequences linearly scales the strength with which a TF binds the target gene. In addition to binding strength following from sequence complementarity, TFs encode an intrinsic binding affinity for promoters *K*_*b*_, representing the structural stability of the TF-DNA binding complex.

TFs can, themselves, be bound to small ligand molecules with binding affinity *K*_*l*_, altering the regulatory effect they exert on downstream genes. These effects are encoded by parameters *eff*_*bound*_ and *eff*_*apo*_ for the ligand-bound and ligand-free state of the TF, respectively, and evolve independently. Ligand binding to TFs is assumed to be a fast process, relative to enzymatic and transcription-translation dynamics, and modeled at quasi steady state. We determine the fraction of TF that is not bound by any of its ligands *L*:

$${W_{apo}} = \prod_{l \in L} \left(1 - \frac{[l]}{[l] + K_{l}}\right) $$

The fraction of time that a TF *τ* in a particular state *σ* (bound or apo) is bound to a particular operator *o*:

$$V_{o} = \frac{[\tau_{\sigma}] \cdot c_{\tau o} \cdot K_{b_{\tau}}}{1 + \sum_{\sigma \in \mathcal{S}} \sum_{{\tau_{\sigma}} \in \mathcal{T}} [{\tau_{\sigma}}] \cdot c_{\tau o} \cdot {K_{b_{\tau}}} } $$

depends on the inherent binding affinity ${K_{b_{\tau }}}$ as well as the sequence complementarity score *c*_*τo*_ between the tf binding motif and the operator sequence [cite Neyfahk]. The binding polynomial in the denominator is the partition function of all TFs $\mathcal {T}$ in any of the states $\mathcal {S}$ that can bind the operator. Note that small declines in the concentration of free TFs due to binding to operators are neglected.

Now, the operator mediated regulation function for any gene is given by

$${Reg} = \sum V_{i} \cdot E_{i} $$

with *V*_*i*_ the fraction of time that the operator is either unbound or bound by a TF in either ligand bound or unbound state and *E*_*i*_ the regulatory effect of that state (1 if unbound or *eff*_*bound*_ or *eff*_*apo*_ when bound by a ligand bound or ligand free TF, respectively). Finally, protein concentrations $[\mathcal {P}]$ are governed by the function:

$$\frac{d[\mathcal{P}]}{dt}={Pr} \cdot {Reg} \cdot {degr} \cdot [\mathcal{P}] $$

where *Pr* is the evolvable parameter *promoter strength* and *degr* a fixed protein degradation rate which is not evolvable.

**Toxicity and death** Virtual Microbe death is a stochastic process depending on a basal death rate, which is potentially increased when internal metabolite concentrations reach a toxic threshold. A cumulative toxic effect is computed over the current life time *τ* of a microbe as

$${e_{tox}} = \sum_{m\in M}{} \int_{t=0}^{\tau} f(m,t) dt $$

for all internal molecules *M*, with

$${\kern32pt}f(m,t) = {max}\left(0, \frac{[m]_{t} - {tox_{m}}}{{tox_{m}}}\right) $$

the toxic effect function for the concentration of molecule *m* at time *t* with toxicity threshold *tox*_*m*_. This toxic effect increases the death rate *d* of microbes starting at the intrinsic death rate *r*

$$d = \frac{{e_{tox}}}{s+{e_{tox}}} \cdot (1-r) + r $$

where *s* scales the toxic effect. Virtual Microbes that survive after an update cycle retain the toxic level they accumulated so far. Apart from toxicity and stochastic death, cells can also starve. When insufficient biomass product is available to keep up the slowly decaying volume of the cell, the cells decrease in volume. If the cell volume drops below a *minimally viable volume*, this cell is automatically for death.

**Reproduction** When an empty grid point is available, the 8 (or less) neighbouring competitors get to compete to reproduce into the grid point. During the ‘in silico serial transfer protocol’ (see below), all cells are continuously mixed, so 8 (or less) random competitors are sampled. When cells compete for reproduction, the cells are ranked according to cell size. The “winner” is then drawn from a roulette wheel with weights proportional to this ranking. Upon reproduction, cell volume is divided equally between parent and offspring, and the genome is copied with mutations (see below). Molecule and protein concentrations remaining constant. Toxic effects built up during the parent’s lifetime do not carry over to offspring.

**Genome and mutations** The genome is a circular list of explicit genes and their promoter region, organised like “pearls on a string”. Genes can be enzymes, transporters, or transcription factors. At birth, the genome is subject to various types of mutations. Large mutations include duplications, deletions, inversions, and translocations of stretches of genes (see Table 1). At the single gene level, point mutations allow all evolvable parameters to mutate individually (see Table 2). Horizontal gene transfer can occur on every time step. Innovations are an abstraction of “HGT from an external (off-grid) source”, and allow randomly parameterised genes to be discovered at any given moment with a low probability.

### Experimental setup

**Metabolic network and wild type evolution**We use a very simple metabolic network with 2 resource metabolites, 1 building block metabolite, and an energy carrier (Fig. 2a). We initialised 16 minimally viable Virtual Microbes, and evolved them for ∼10.000-15.000 generations in fluctuating resource conditions by applying random fluctuations of the influx rates for the A and the C resource. Because the rate of influx for the two resource metabolites fluctuates between very high (10^−1^) and very low values (10^−5^), conditions can be very poor, very rich, and/or potentially toxic. To avoid total extinction, we subdivided the 40x40 grid into four 20x20 subspaces, in which these fluctuations are independent (see Fig. 2b). Note however that these subspaces do not impede diffusion and reproduction, but merely define the rate at which resources flux into different positions on the grid. In this study, the microbes do not migrate during their lifetime. These conditions, summarized in Table 3, aim to simulate natural resource fluctuations, evolving what we call “wild types” (WTs) of Virtual Microbes. (see Additional file 1: Section S1).

The initial population consists of cells that have 3 enzymes, 3 pumps, and 5 transcription factors. All these proteins are randomly parameterized, meaning that these proteins are unlikely to have good binding affinities and catalytic rates. The amount of building block required to grow and produce protein is therefor very minimal in the early stages of evolution, and is increased up to a fixed level as the Virtual Microbes become more productive over time.

**In silico serial transfer protocol** We mimic a serial transfer protocol like by taking our evolved WTs and – instead of fluctuating the resource conditions – periodically supplying a strong pulse of both the A- and the C-resource. While WTs are evolved in a spatial setting where resources flux in and out of the system, we here mix all cells and resources continuously and fully close the system, meaning no metabolites wash in or out of the system during the daily cycle. To apply strong bottlenecks while at the same time allowing for sufficient growth, we increased the size of the grid from 40x40 to 70x70. We then dilute population approximately tenfold, transferring 500 cells to the next cycle. Horizontal gene transfer between cells was disabled to represent the modified (asexual) *Escherichia coli* REL606 clone that is used in the LTEE [1]. Finally, as the strong bottlenecks cause more genetic drift in our small populations than in the WT evolution, we found it necessary to dial back the mutation rates for the evolution of WTs to 30% to avoid over-exploiting mutants from appearing to easily (see Table 1). Other parameters of the serial transfer protocol are listed in Table 3.

**Growth rate and yield measurements**

Yield was approximated by taking sum of all cell volumes. We measured yield both within a single serial transfer cycle (“daily yield”), and as the extended yield when we tested for long-term survival. As all WTs had slightly temporal growth rate dynamics, we estimated the growth rates as the average building block production during the first half of the protocol.

**Characterising coexistence**

Using the neutral lineage markers (also see Additional file 1: Figure S8), we manually characterised coexistence by looking at the dynamics of neutral lineage markers. When two neutral markers had relatively stable frequencies as visualised in Fig. 5b-c for at least 10.000 time steps (approximately 100 generations), it was scored as coexistence. Sometimes coexistence did not last until the end of the simulation, which we refer to as quasi-stable coexistence.

**Further configuration of Virtual Microbes** Apart from the parameters within the confines of this article (Tables 1, 2, 3 and 4), we have used the default settings for Virtual Microbes release 0.1.4, with the configuration files provided in Additional file 1: Section S2. Further details on the model and parametrisation are available online https://bitbucket.org/thocu/virtual-microbes

## Supplementary information

**Additional file 1** Supplementary materials.

## Data Availability

The full python module of Virtual Microbes is publicly available via PyPi. The code is available online on https://bitbucket.org/thocu/virtual-microbes. Further help with installation, instructions on how to use Virtual Microbes, and full documentation of the methods, is available on https://www.virtualmicrobes.com. As the data to support this study is fully computer generated, and consists of quite a large set of files, we felt it unnecessary and unhelpful to make the data available online. However, all the data that support this study are reproduced using Virtual Microbes 0.1.4 and the configuration from the Additional file 1. Finally, the corresponding author is available for help with the software.

## Abbreviations

GRN: Gene regulatory network (plural: GRNs)
LTEE: Long term evolution experiment (first published by R Lenski, 1991)
TF: Transcription factor (plural: TFs)
WT: wild type (plural: WTs)
