# Supplementary material for: Trusting the hand that feeds: microbes evolve to anticipate a serial transfer protocol as individuals or collectives
Source: BMC Evol Biol. 2019 Nov 4;19:201. doi: 10.1186/s12862-019-1512-2 (PMC6829849; doi:10.1186/s12862-019-1512-2)
Supplement: Supplementary file 1 — Additional file 1 Supplementary materials. [file 12862_2019_1512_MOESM1_ESM.pdf]

# Supplementary materials - S1: Evolution of Virtual Microbes wild types

### Convergent and divergent evolution in Virtual Microbe wild types

In the evolution of our WTs we observed strong convergence as well as divergence in the metabolic and gene regulatory networks that evolved. Because the evolved populations consist of a rich mix of different genotypes, we here describe the WTs by profiling the gene repertoires and GRNs at the end of the simulation (∼10.000 generations). For this, we took 20 (maximally unrelated) individuals from the evolved populations and determined the consensus metabolism (Figure S1A). While there is some diversity in the metabolic networks across WTs, the shared gene repertoire constitutes a metabolic network that forms a metabolic cycle complemented with resource importers and an exporter for the C metabolite (Figure S1B). We observed that the discovery of both the metabolic cycle as well as the exporter favour survival, as it coincides with an increase in population size and a decrease in the number of generations per time step (Figure S4). Note that in Virtual Microbes survival is improved by avoiding toxic effects of high metabolite concentrations and by only investing in growth when conditions are favourable for growth. The latter can be done via gene regulatory networks that respond to the quality of the environment, but we also found forms of metabolic regulation where microbes accurately fine-tuned kinetic parameters to automatically maintain homeostasis.

Although the shared gene repertoire from Figure S1B does not contain transcription factors (TFs), all of the 16 WTs have at least one type of TF. These TFs can constitutively repress or activate certain genes, or can respond to environmental conditions by binding to a ligand molecule. The latter response depends on the kinetic properties of the TFs and the properties of the genes which they regulate, all of which are evolvable (see Table S1). To get a better overview of how the WTs respond to environmental stimuli we therefore chose to directly measure the gene expression levels in a variety of different resource concentrations (displayed for 6 WTs in Figure S2). On the level of these GRNs, and their sensitivity the environment, we clearly see signs of strong divergent evolution. Note however, that the *effect* on the importer and exporter proteins seems very similar between WTs with different networks, showing that similar responses can be encoded by different GRNs. Finally, as seen in these graphs, some WTs have no response to environmental stimuli. We found that these non-regulating WTs are equally “fit”, in that they have the same rates of building block production and death rates (see Figure S3). However, the majority (11/16) WTs evolved clear regulatory mechanisms.

In short, during the *de novo* evolution of Virtual Microbe WTs, some evolved features seem highly predictable. Namely, all have evolved the metabolic cycle, all express both resource importer proteins, and all but one WT have a C-exporter. On

the other hand, regulatory mechanisms and some of the secondary reactions display considerable diversity. Note that this divergence cannot be explained by differences in initial conditions or fluctuations in resource concentrations, because the WTs only differ with respect to the mutations that have happened in their evolutionary history. However, as shown in the main text, these differences have a profound effect on further evolution.

**Table S1 - Important parameters for TFs for an environmental response**

| Property TF | Description |
|---|---|
| Expression TF | The TF itself needs to be sufficiently expressed |
| Binding motif | The binary binding motif (10 bits) must have a sufficient match to the operator sequences of genes (50 bits) in order to affect their expression |
| $K_{ligand}$ | If the binding constant to the ligand $K_{ligand}$ is not in range of the observed concentration of metabolites, the TF will always have the same (up or down) regulatory effect, regardless of the environment or internal concentrations. |
| Effect of ligand | The ligand-bound and ligand-unbound regulatory effects of TFs need to be different to effectively change expression of genes given any environmental stimulus |

**Table S2 - Anticipation and polymorphisms are also observed when changing in the serial transfer protocol** For different transfer times, dilutions, and resources concentrations, seven WTs (11-16, and WT07 from Figure ?? from the main text) have been tested for the anticipation effect and polymorphisms. Note that anticipation is not tested by prolonging the cycle (like in the main text), but by comparing the patterns in cell cycle dynamics with those from Figure 3 in the main text. If a clear decrease in cell volume was observed at the end of the cycle, it was scored as anticipation. Polymorphisms are scored as defined in the methods.

textbf

| WT | Anticipation (Yes/No) | Polymorph (Yes,No,Quasi) |
|---|---|---|
| 11 | Y | N |
| 12 | Y | N |
| 13 | Y | Q |
| 14 | Y | N |
| 15 | Y | Q |
| 16 | Y | Q |
| 07 | Y | N |

textbf

| WT | Anticipation (Yes/No) | Polymorph (Yes,No,Quasi) |
|---|---|---|
| 11 | Y | N |
| 12 | Y | N |
| 13 | Y | Y |
| 14 | Y | N |
| 15 | Y | N |
| 16 | Y | Q |
| 07 | Y | N |

# Supplementary materials - S2: Virtual Microbe configuration

The evolution of these WTs was done with Virtual Microbes version 0.1.4 which is publicly available as a Python package. Complete documentation on the methods is publicly available on http://bitbucket.org/thocu/virtualmicrobes. For this study, we used the configuration below. We removed options that are not relevant for reproducibility (*e.g.* memory-limit, thread-count, data-storage-frequency etc.) or are default (*e.g.* universal-mutation-rate scaling=1.0) To reproduce these results with the newer versions of Virtual Microbes (0.2.4 as of January 2019), feel free to contact the corresponding author if help is required.

```
virtualmicrobes.py @reggen.cfg - evo @reg-evo.cfg --name My_WT_Vmicrobe
```

general_options.cfg:

```
--base-death-rate 0.01
--mutation-rates
chrom_dup=0.0
chrom_del=0.0
chrom_fiss=0.0
chrom_fuse=0.0
point_mutation=0.005
tandem_dup=0.005
stretch_del=0.005
stretch_invert=0.005
stretch_translocate=0.005
stretch_exp_lambda=0.3
external_hgt=0.0002
internal_hgt=0.002
regulatory_mutation=0.005
reg_stretch_exp_lambda=0.1
--point-mutation-ratios
ligand_class=0.1
exporting=0.1
--rand-gene-params
base=10
lower=-1.0
upper=1.0
--mutation-param-space
base=10
lower=-0.5
upper=0.5
min=0.01
max=10.
--max-historic-max 0.1
--growth-rate-scaling 1
--competition-scaling 1
--selection-pressure historic_window_scaled
--historic-production-window 1000
--scale-prod-hist-to-pop
--small-mol-diff-const 0.02
--prot-degr-const 0.7
--transporter-membrane-occupancy .1
--influx-range
base=10
lower=-1.0
upper=-5.0
--fluctuate-frequencies 0,0.01
--init-external-conc 0.
--small-mol-ext-degr-const 1e-2
--bb-ext-degr-const 1e-1
--ene-ext-degr-const 5e-1
--transcription-cost 0.002
--energy-transcription-scaling 0.01
--spill-conc-factor 1.
--v-max-growth 1
--min-bind-score 0.85
--per-grid-cell-volume 8
--enzyme-volume-occupancy 3
--grid-sub-div row=2,col=2
--sub-env-part-influx 1.0
--grid-cols 40
--grid-rows 40
```

evosim_options.cfg:

```
--duration 1000000
--env-rand-seed 87
--reproduce-size-proportional
```

```
--cell-init-volume 1.5
--cell-growth-rate 2.0
--cell-shrink-rate 0.6
--cell-growth-cost 0.2
--cell-division-volume 2.
--init-prot-mol-conc .1
--max-cell-volume 5
--nr-resource-classes 3
--nr-energy-classes 1
--ene-energy-range 1,1
--res-energy-range 2,10
--nr-building-blocks 1
--building-block-stois 1,1
--nr-cell-building-blocks 1
--mol-per-ene-class 1
--mol-per-res-class 1
--nr-cat-reactions 3
--nr-ana-reactions 3
--max-nr-cat-products 2
--min-cat-energy 1,3
--max-nr-ana-products 1
--nr-ana-reactants 2
--chromosome-compositions tf=0,enz=1,pump=1
--binding-seq-len 10
--operator-seq-len 50
--prioritize-influxed-metabolism
--init-prot-mol-conc 0.01
--degradation-variance-shape 100
--no-toxicity-variance-shape
--toxicity 0.2
--toxic-building-blocks
--toxicity-scaling 1000
--tf-binding-cooperativity 2
--homeostatic-bb-scaling 1
--high-energy-bbs
--prioritize-energy-rich-influx
```

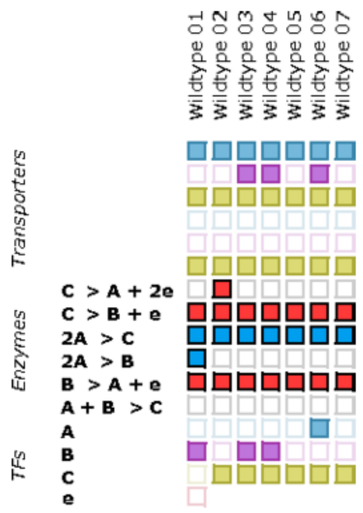


**Figure S1 The evolved gene repertoires for all 16 WTs** The gene repertoires of WTs (20 maximally unrelated individuals) is displayed for all 16 replicate simulations after 1.000.000 time steps. Rows represent the different types of proteins (transporters, enzymes and TFs), and the columns the gene repertoires. Note that the presence of a gene does not imply it is functional, since properties such as $K_s$ and $V_max$ might be poorly parameterised. Genes found in less than 4 cells or genes with low concentrations (*i.e.* low expression) were omitted. The seperate column depicts the genes found in at least 90% of WTs.

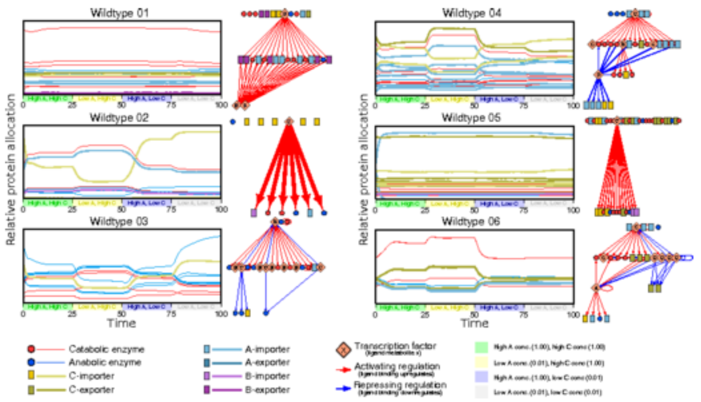


**Figure S2 WTs have great diversity of transcriptional regulation, and not all respond to changing resource concentrations** The graphs shows how the protein allocation shift to imposed changes in resource concentration of the environment. Single clones from the WTs were taken and resource concentrations of the A- and C-resource were varied from low (0.01) to high (1.0). The gene regulatory network responsible for these changes is displayed next to each graph. The colours for different enzymes are as displayed in the legend. Thicker arrows in the gene regulatory network represent higher expression levels of the transcription factors. Genes with very low expression levels were omitted for clarity.

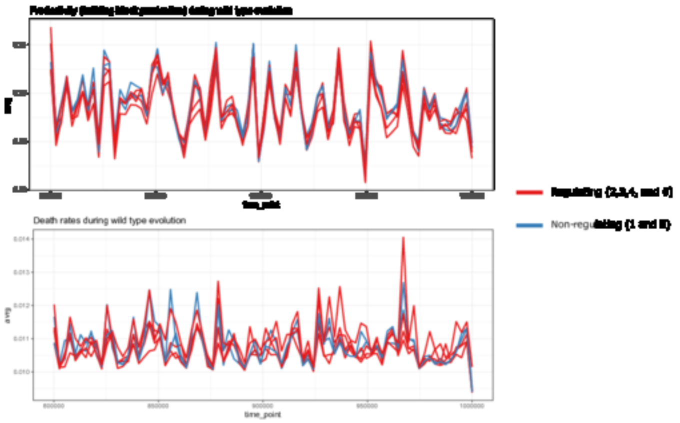


**Figure S3 Different WTs have similar building block production rates and death rates.** For 6 WTs, the last 2000 generation of WT evolution did not show any differences in “fitness”, which is approximated by the rates of building block production and death. Furthermore, regulating WTs showed not difference compared to non-regulating WTs.

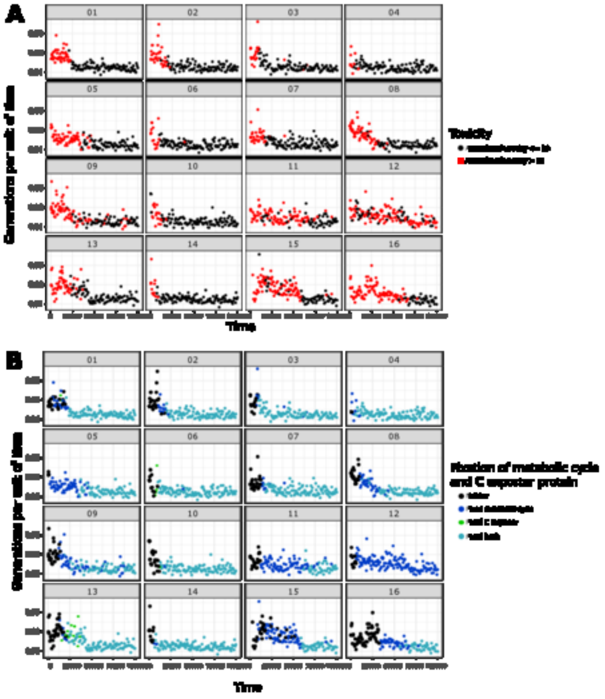


**Figure S4 Decrease in number of generations per time step for WTs coincides with a decrease in toxicity and/or fixation of a metabolic cycle** Decrease in number of generations per time step coincides with a decrease in toxicity accumulation and/or fixation of a metabolic cycle. Every dot represents an average over a 100 generations of simulation. For panel A, red dots represent a toxicity level above which death rate is increased at least threefold (toxicity $> 20$), and black dots represent the lower toxic levels (toxicity $<= 20$). In panel B, the cyan dots represent the fixation of both the C-exporter and the metabolic cycle, green dots the fixation of only the C-exporter, blue dots the fixation of the metabolic cycle, and black dots the fixation of neither of these features.

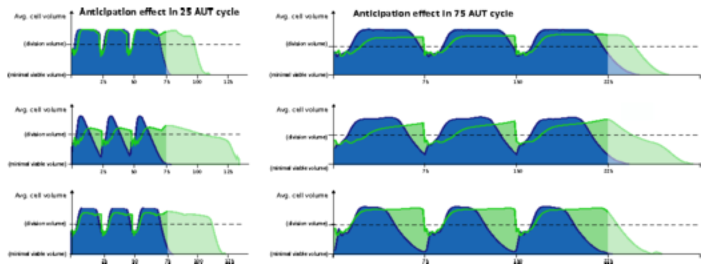


**Figure S5 The anticipation effect is present after evolving in shorter (left) and longer (right) time intervals.**

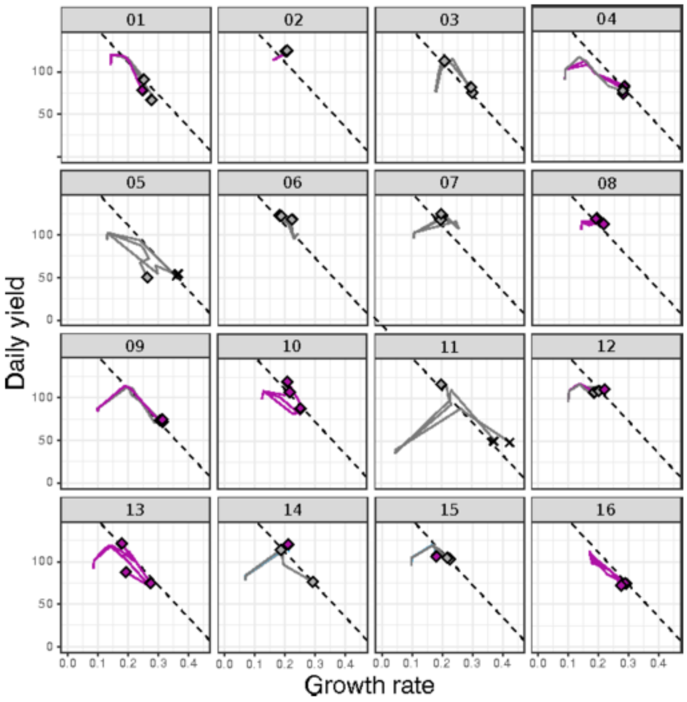


**Figure S6 Three replicate serial transfer experiments for all 16 WTs.** This image is shows all the data from the examples from Figure ??.

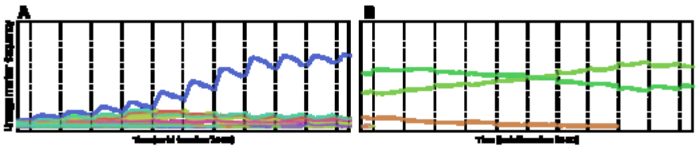

**Figure S7 Dynamics of different invading mutants** A) Neutral lineage markers linked to mutations which cause high growth rates but poor survival increase in frequency early, but are losing in frequency during stationary phase. B) Neutral lineage markers linked to mutations that have higher growth rates without trading off against survival show no such temporal fitness effect.

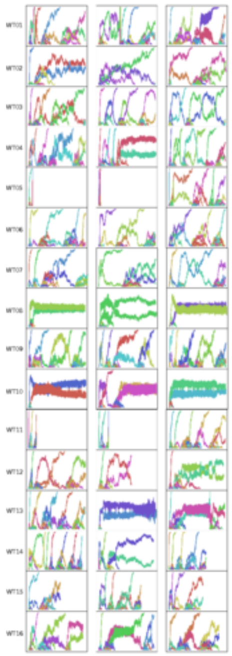

**Figure S8 Dynamics of lineage markers in all simulations** The neutral lineage markers for all 3 replicates of all WTs is shown, from which the coexistence was inferred (see methods).

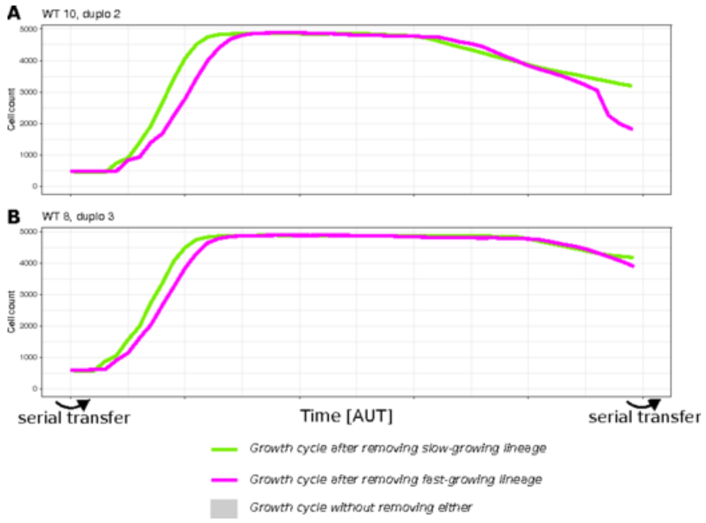


**Figure S9 Lineages based on resource specialisation (top) and growth vs. yield specialisation (bottom) perform better in the presence of the other** In the upper panel, two resource specialists over-exploit their resource in the absence of the other. In the bottom panel, without evident resource specialisation, both lineages have increased toxicity (given that this happens through enzymatic changes, this is likely due to changes in fluxes), which accumulates to significant levels near the end of the cycle. This figure thus shows that lineages can grow dependent on one another, as they are part of each-others environment.
